# Flexible and stretchable chromatic fibers with high sensing reversibility†
†Electronic supplementary information (ESI) available. See DOI: 10.1039/c6sc00414h


**DOI:** 10.1039/c6sc00414h

**Published:** 2016-04-14

**Authors:** Xin Lu, Zhidong Zhang, Xuemei Sun, Peining Chen, Jing Zhang, Hui Guo, Zhengzhong Shao, Huisheng Peng

**Affiliations:** a State Key Laboratory of Molecular Engineering of Polymers , Department of Macromolecular Science , Laboratory of Advanced Materials , Fudan University , Shanghai 200438 , China . Email: sunxm@fudan.edu.cn ; Email: zzshao@fudan.edu.cn

## Abstract

Chromatic polymers, such as polydiacetylene (PDA) that display color changes under stimulations, have been widely explored as sensors and displays. However, the PDA-based materials are generally rigid and irreversible in the chromatic transition. Herein, a flexible and stretchable PDA composite fiber is produced by incorporating peptide-modified PDA into aligned carbon nanotubes on an elastic fiber substrate. It performs a rapid and reversible chromatic transition in response to electrical current that can be repeated for 1000 cycles without fatigue. Due to their high flexibility and stretchability, these chromatic fibers can be integrated into different patterns and woven into smart textiles for displaying and sensing applications.

## Abstract 2

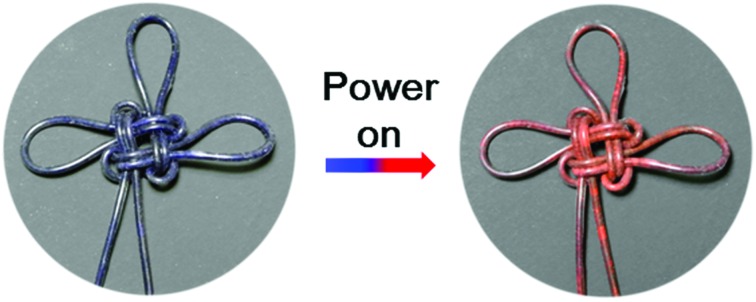
Flexible, stretchable and highly reversible electrothermal chromatic fibers are developed from aligned carbon nanotubes and peptide-modified polydiacetylene.

## Introduction

Materials that can change colors under environmental stimulations have been widely explored due to their promising applications in sensing, detecting and displaying.^1^–^6^ Among them, conjugated polymers such as polydiacetylene (PDA) have been mostly investigated for contrast chromatic transitions typically between blue and red due to the conformation change of the PDA backbone in response to heat, pH, organic solvent, ligand–receptor interaction and mechanical stress.^7^–^12^ The sensing PDA materials that had been made into powders and films were typically rigid and robust. However, for next-generation portable and wearable electronics, the electronic and sensing devices need to be flexible and stretchable.^13^

Chromatic transitions of PDA materials are usually slow as it takes time for the stimuli transport to give enough energy to the side chains and induce the conformation change of the PDA backbone. For instance, the most common heat and solvent induced color changes of PDA were on the level of seconds to minutes.^10^,^14^ Furthermore, the color changes are mostly irreversible after the removal of stimuli because the interactions between the side chains are typically not strong enough to restore the backbone conformation in PDA. To this end, various functional groups providing plenty of hydrogen bonding, π–π interaction and even chemical bonds were non-covalently and covalently incorporated into the side chains of PDA to improve the capability to reversibly switch the backbone conformation by forming ordered and stable assemblies.^2^,^15^–^20^ In addition, second phases such as polymers, silicas and metal oxides have been introduced to prepare composite materials.^21^–^25^ However, most of the modified PDAs with a reversible color transition were induced by heating and cooling cycles, which would be inconvenient in some practical areas. Recently, carbon nanotubes (CNTs) were introduced to prepare conducting PDA composites.^26^ A new stimulus of electric current was passed through the composite material to induce a rapid color change in less than one second. However, to summarize, the chromatic transition was reversible for only tens of cycles which needs to be greatly enhanced.

A few flexible electrochromatic devices have also been made from other conjugated polymers to meet the requirements. Electrochromatic fibers were reported to reversibly switch colors through the incorporation of several conducting polymers, while the stretchability was limited by using a metal wire as the electrode.^27^ Flexible electrochromatic fiber-shaped supercapacitors were also explored with the introduction of polyaniline, and they showed chromatic transitions during the charge–discharge process for thousands of cycles.^28^ However, the complex structure and the use of an electrolyte have hindered their practical application in wearable and portable electronics.

**Scheme 1:**
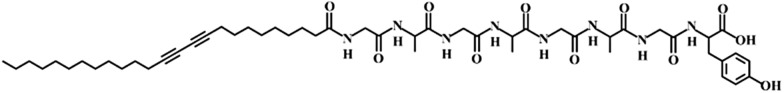
Molecular structure of the peptide modified DA monomer.

Here, a flexible and stretchable PDA based fiber is created to display rapid and highly reversible electrothermal chromatic transitions. The flexible and stretchable capability is mainly contributed from the elastic polymer fiber substrate. Aligned CNT sheets are wound on the polymer fiber to offer the conducting property. A peptide segment of Gly–Ala–Gly–Ala–Gly–Ala–Gly–Tyr (GAGAGAGY) is incorporated into the PDA side chains with multiple hydrogen bonds for a high reversibility of chromatic transitions (Scheme 1).^29^ Upon passing an electric current through the material, the elastic CNT/PDA composite fiber changes color in milliseconds, and the electrothermal chromatic transitions can be repeated for 1000 cycles without obvious fatigue. Also, the rapid and reversible electrochromism of the composite fiber is maintained well under bending and stretching.

## Experimental

### Materials

Poly(dimethylsiloxane) (PDMS) elastomer was obtained from Dow Corning (Sylgard 184). Ag nanowires dispersed in ethanol (CST-NW-S50) were ordered from Suzhou ColdStones Technology Co., Ltd. Silicone liquid (CAF-3) was purchased from Bluestar Silicones France Co., Ltd. Ethanol (purity 99.7%) was obtained from Sinapharm Chemical Reagent Co., Ltd. Fmoc–Tyr(*t*Bu)–2-chlorotrityl resin, Fmoc–Gly–OH, Fmoc–Ala–OH, HOBT, TBTU and DIPEA were obtained from GL Biochem Co., Ltd. (Shanghai, China). 10,12-Pentacosadiynoic acid was purchased from Alfa-Aesar Co., Ltd. All other regents, compounds, and chemicals were obtained from commercial suppliers and used without further purification.

### Preparation of the composite fiber

The precursor of the elastomer prepolymer and curing agent at a weight ratio of 10/1 was injected into a shrinkable tube (diameter of ∼1.8 mm), followed by curing at 80 °C for 2 h. The PDMS fiber was obtained by removing the outer tube. Aligned CNT sheets were drawn from a spinnable CNT array synthesized by chemical vapor deposition.^4^,^26^ Due to the high van der Waals force, they were closely wrapped onto the PDMS fiber with an angle of 70° to obtain conductive fiber substrates by a rotating translation method.^30^ A silver nanowire solution dispersed in ethanol with a concentration of 5 mg mL^–1^ was dip-coated onto the conductive fiber, followed by wrapping with another layer of CNT sheet with an angle of 70°. The monomer C_25_-GAGAGAGY (Scheme 1) was prepared by grafting octapeptide of GAGAGAGY onto the side chains of 10,12-pentacosadiynoic acid, and the synthetic details are described elsewhere.^29^ The monomer (100 mg) was then dissolved in NaOH aqueous solution (5 mL, 0.01 M) at 60 °C. After the solution was cooled down to room temperature, concentrated hydrochloric acid was added dropwise until the pH was ∼6 to make the solution gel. Then, 5 mL ethanol was added to the solution to improve its solvent affinity to CNTs.^31^ The blue PDA fiber was obtained by uniformly dip-coating a gelled monomer solution onto the conductive fiber, followed by topological polymerization under UV light (wavelength of 254 nm) at a distance of 5 cm for 3 min. Finally, transparent silicone was introduced to encapsulate the PDA fiber and it was cured at room temperature for 24 h. The thickness of the silicone was about 70 μm. Electric currents up to 80 mA were investigated, and an electric current of 8.1 mA was generally used, unless specified otherwise.

### Characterization

Scanning electron microscope (SEM) images were recorded using a field emission microscope (Hitachi S-4800, Japan) operated at 1 kV. Samples for SEM were sprayed with a thin layer of gold before observation. The electrical conductivity was obtained from a Keithley 2400 Source Meter and VICTOR VC9807A+ digital multimeter. The current was also supplied by a Keithley 2400 Source Meter. Electrical properties during stretching were characterized using a Keithley 2400 Source Meter together with a Shanghai Hengyi Universal Testing Instrument. Photographs were taken by a digital camera (Nikon J1, Tokyo, Japan). Absorption spectra were obtained using a miniature fiber optic spectrometer (Idealoptics PG2000-pro, China) installed in an optical microscope (Olympus BX51, Japan).

## Results and discussion

To obtain the electrothermal chromatic fiber, a flexible and stretchable conductive fiber was firstly prepared by winding the aligned CNT sheets around an elastic polydimethylsiloxane (PDMS) fiber (Fig. S1a†).^30^ To improve the electrical conductivity of the composite fiber, silver nanowires were incorporated onto the aligned CNT sheets through a dip-coating process. Another CNT sheet was then wound around the silver nanowire-coated fiber to stabilize the silver nanowire. The sandwiched silver nanowires between the two CNT sheets were uniformly distributed with an interlinked connection and compactly contacted with the CNTs (Fig. S1b†). As expected, the CNTs remained highly aligned on the elastic fiber before and after stretching (Fig. S1c†).^30^ The as-prepared composite fibers were flexible and stretchable with an electrical resistivity of ∼60 Ω cm^–1^. No obvious fatigue in structure was observed for the conductive fiber after stretching for 1000 cycles with a strain of 50% (Fig. S2†). Also, the resistance exhibited little change when they were bent from 0 to 180° and stretched up to 80%. In addition, the resistance remained stable after bending at 90° and stretching by 50% for over 1000 cycles (Fig. S3†).

**Fig. 1:**
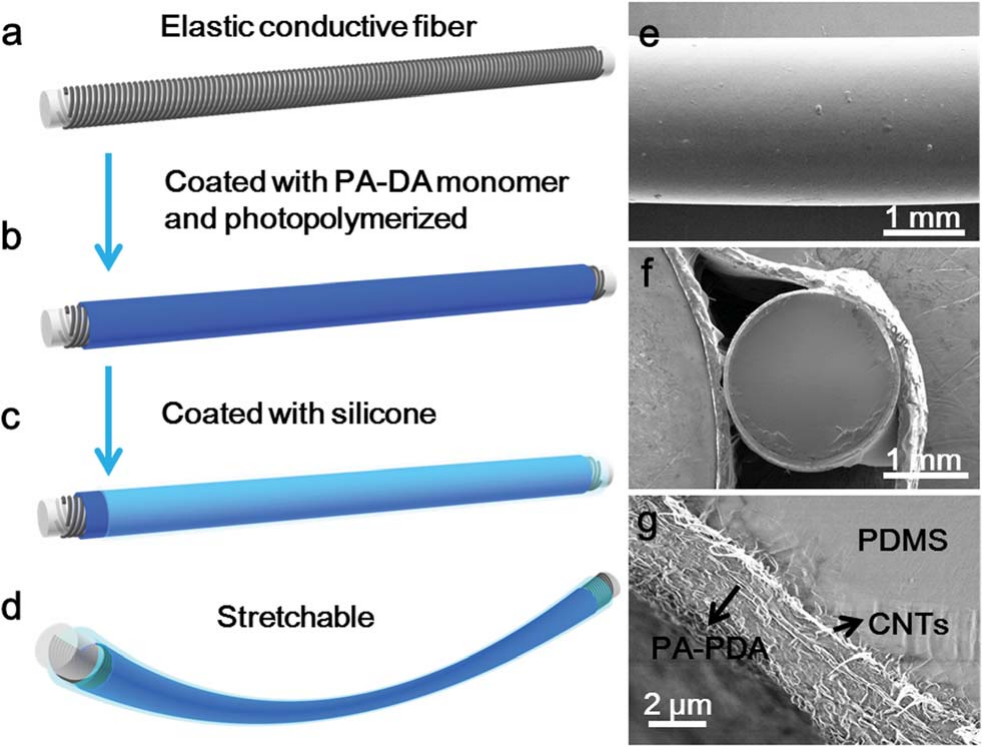
Preparation and structure of the stretchable electrothermal chromatic fiber. (a–d) Schematic illustration of the preparation process. (e) Side view scanning electron microscope (SEM) image of a stretchable electrothermal chromatic fiber. (f and g) SEM images of the cross-sectional structure at low and high magnifications.

For a typical preparation of the electrothermal chromatic fiber (Fig. 1a–c), the peptide modified DA monomer solution was first dip-coated onto the elastic conductive fiber, followed by topological polymerization under UV light. The composite fiber could be further encapsulated by a transparent silicone layer for the convenience of characterizations and protection from electric leakage. The chromatic fiber was expected to be stretchable (Fig. 1d), which will be discussed later. Fig. 1e shows that the final fiber has a clear and neat surface with the composite wrapped inside. From the cross-sectional view, the fiber held a core-sheath structure (Fig. S4†), and the aligned CNT/PDA composite was sandwiched between the inner and outer elastic silicone with a thickness of ∼2 μm (Fig. 1f and g).

**Fig. 2:**
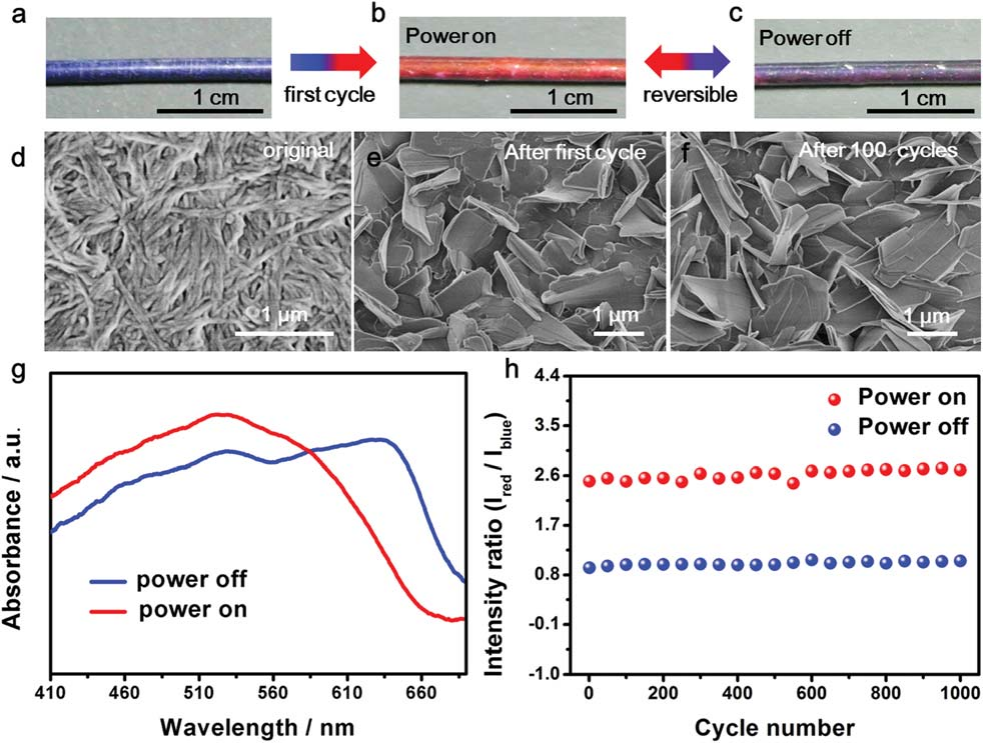
Electrothermal chromatic behavior of the composite fiber. (a–c) Photographs of the electrothermal chromatic composite fiber in the first power on/off cycle; (b and c) are reversible in the following cycles. (d–f) SEM images of PDA in the composite fiber. PDA formed nanofibers at the original state (d), changed to microsheets after the first power on/off cycle (e) and maintained the morphology of the microsheets after the 100th cycle (f). (g) UV-vis spectra of the composite fiber at the power “off” and “on” states. (h) The intensity ratios of red to blue peaks at the “off” and “on” states for 1000 cycles.

The resulting composite fiber appeared as blue (Fig. 2a) and changed to red (Fig. 2b) upon the passing of electric current. The response time depended on the passed electrical current, *e.g.*, typically 0.2 s at 80 mA. The color was recovered in 20 s. The red fiber switched to dark purple after removal of the current (Fig. 2c) and transited to red when the current was provided again. The electrothermal chromatic transition between dark purple and red was reversible in the following cycles (Fig. 2b and c). The morphologies of the PDA at different states were observed using a scanning electron microscope. The PDA originally assembled into nanofibers (Fig. 2d) due to the strong hydrogen-bonding effect between the neighboring side chains. After the first cycle of electric current, they recrystallized into microsheets (Fig. 2e), which may account for the color irreversibility in the first cycle. In the following cycles, such as after 100 cycles, the PDA still exhibited a microsheet structure (Fig. 2f), demonstrating a high stability in accordance with the reversible color transition. The variation of electrical resistance of the fiber also verified the stable recrystallized phenomenon (Fig. S5†). When an electrical current was passed through the fiber at the second cycle, the resistance decreased gradually and then reached a stable state. After removal of the electrical current, the resistance recovered to the original value. These changes were also in agreement with the previous thermochromatic behavior studied with differential scanning calorimetry (DSC), in which the endothermic peak located at 60 °C showed a difference between the first and second cycles, and it could be completely reversible from the second cycle.^29^

UV-vis absorption spectra were further used to quantitatively trace the color changes. The two main peaks at ∼640 nm and ∼525 nm corresponded to the blue and red phases of PDA, respectively. When the currents were initially increased, the original peak intensities at 640 nm gradually decreased, while a new peak at 525 nm appeared and increased (Fig. S6a†), in accordance with the color change from blue to red. After removal of the current, the absorption spectra didn't completely recover due to the partially irreversible conformational transition of PDA,^29^ but reached a reversible equilibrium with two coexisting peaks of 640 and 525 nm (Fig. S6b†). The coexistence of blue and red phases acted on the fiber, resulting in a mixed color of dark purple. When the currents were applied at the second cycle, the two peaks of 640 and 525 nm turned into one main peak at 525 nm, reflecting the color change from dark purple to red (Fig. 2g). From the second cycle, the spectrum and color completely reversed back after removal of the current. Therefore, the reversibility of the chromism was mainly studied from the second cycle with a current of 8.1 mA. The intensity ratio of the peaks at 525 nm to 640 nm was used to study the chromatic reversibility of the fiber. The ratios at red and dark purple states were calculated as 2.55 and 0.93, respectively. Fig. 2h shows the change of intensity ratios over 1000 cycles with and without the passing of electric current. They varied by less than 10%, indicating a decent reversibility. Additionally, the chromatic cycles remained reversible with increasing currents up to 25.2 mA, and they varied for different recovered states (Fig. S7†). This phenomenon was mainly caused by partially irreversible conformational transitions at the first cycle under different electrothermal conditions.^29^ The reversible current depended on the duration time. When the current was higher than 25.2 mA and maintained for 25 s, the color could not completely recover to the original state. However, the chromatic transition was reversible even at a higher current of 80 mA for a shorter duration of 0.2 s.

PDAs were previously reported to show a chromatic transition due to the electrically induced polarization, but with a poor color contrast on a black CNT substrate.^26^ They also showed a thermochromatic behavior; however, it remained challenging to make a remote control for their color changes, which is important in many electronic applications.^29^ Here, the chromatic fiber was designed to have a color change dominated by an electrothermal effect, with a good performance. As shown in Fig. 1g, the inner conductive PDA/CNT layer first formed with a thickness of ∼150 nm, followed by a coating of a much thicker bare PDA layer of ∼2 μm. The color appearance was therefore mainly contributed by the outermost PDA layer and was further verified by Raman spectroscopy (Fig. S8†). For the inner PDA layer in contact with the CNT, due to the interactions between them, the characteristic peaks for both CNT and PDA were shifted, *i.e.*, from 1578 and 2649 cm^–1^ to 1581 and 2657 cm^–1^ for CNT and from 1454 and 2085 cm^–1^ to 1459 and 2092 cm^–1^ for PDA. However, only the characteristic peaks of PDA existed for the composite fiber. In other words, the color change of the composite fiber was produced by the outermost PDA, mainly based on an electrothermal effect. The impact of fiber size and monomer concentration was also carefully investigated. A PDMS fiber with a diameter of 1.2 mm was used to replace the previous one with a diameter of 1.8 mm as the elastic substrate. Due to a resistance increase of ∼42%, the transition current decreased to 6.8 mA. In addition, as the resistance increased by ∼14% and ∼46%, the fibers from monomer solutions with concentrations of 15 and 20 mg mL^–1^ exhibited transition currents of 7.6 and 6.7 mA, respectively. Moreover, due to the rapid heating by the aligned CNTs, the color change of PDA was also fast.^29^

When an electric current passed through the composite fiber, the PDA was rapidly heated by the underlying CNTs and silver nanowires as nanoheaters (Fig. S9†). For instance, the temperature of the composite fiber was about 70 °C when appling an electric current of 8.1 mA, which was higher than the thermochromatic transition temperature of PDA. The chromatic transition of PDA resulted from the change of the conjugation length of the backbone perturbed by the side chain under external stimulations.^32^–^34^ The strong interactions generated by plenty of hydrogen bonds among the side chains offer the PDA a great capability to recover the backbone conformation for the chromatic reversibility.^31^,^35^,^36^ The peptide side chains could assemble into β-sheets with stable conformations, which enhances the reversibility of the PDA backbone.^29^,^37^ The introduction of the CNTs also helps the regular recrystallization of the PDA to form larger, and separated, microsheets (Fig. 2f) compared to the bare PDA (Fig. S10†), which further improves the stability of the chromatic property.

**Fig. 3:**
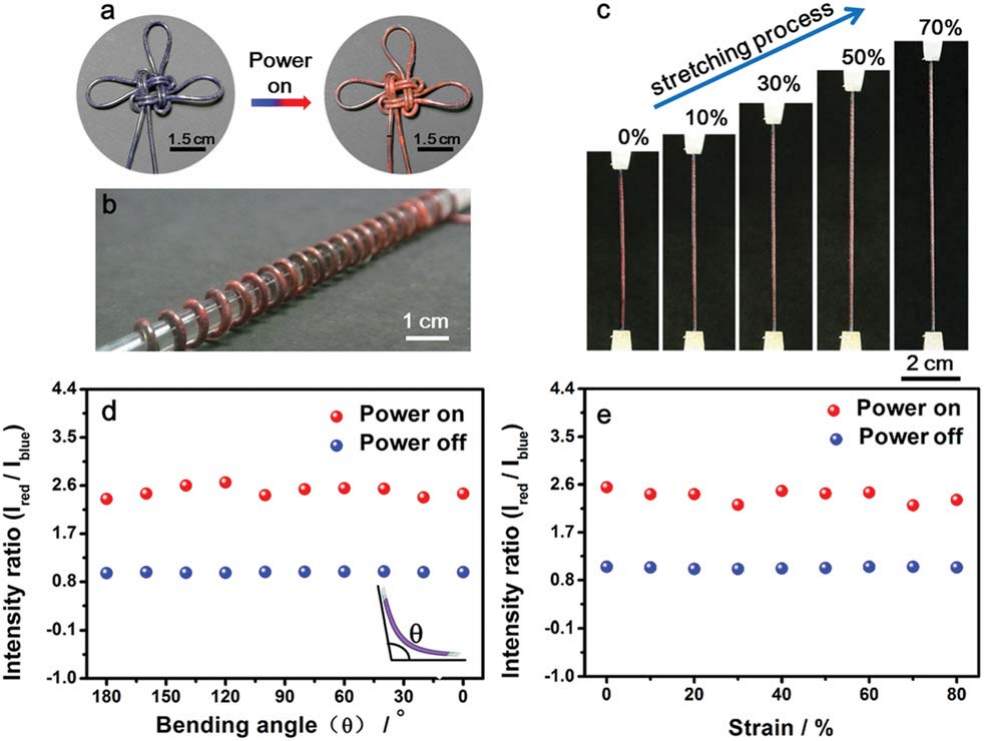
Flexible and stretchable characterizations of the fiber. (a) An electrothermal chromatic Chinese knot prepared from the electrothermal chromatic fiber. (b) An electrothermal chromatic fiber wound on a glass rod (diameter of 1 cm). (c) *In situ* photographs during stretching. (d and e) Dependence of the intensity on the bending angle and strain, respectively. Here *I*_red_ and *I*_blue_ correspond to the intensities of red and blue peaks, respectively.

Benefiting from the elasticity of the PDMS substrate and the resistance stability of the conductive layer, the electrothermal chromatic fibers were highly flexible and stretchable. For instance, the reversible chromatic transitions were well maintained under and after bending, twisting and winding onto substrates (Fig. 3a and b). Similarly, they had been also well maintained under stretching (Fig. 3c). Both the structure and resistance of the composite fiber remained almost unchanged before and after bending or stretching (Fig. S11†), which was important for a stable performance during use. The flexible and stretchable properties were further quantitatively characterized by the intensity ratio of red to blue peaks at different bending and stretching states. No obvious decrease in the intensity ratio was observed with decreasing bending angles from 180° to 0° (inset, Fig. 3d). The intensity ratios remained almost unchanged when the fiber was stretched up to 80% (Fig. 3e). The intensity ratios remained stable even after stretching by 50% for 1000 cycles (Fig. S12†).

**Fig. 4:**
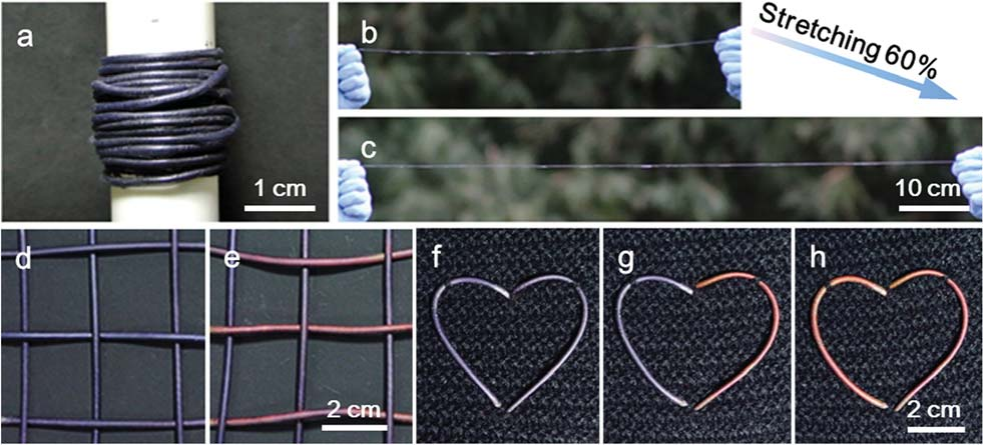
Photographs of the electrothermal chromatic fibers and woven smart fabric patterns. (a) Long electrothermal chromatic fiber being coiled into a spool. (b and c) Photographs of an electrothermal chromatic fiber before and after stretching by 60%, respectively. (d and e) Photographs of an electrothermal chromatic fabric with the transverse fibers changed from blue to red. (f–h) Electrothermal chromatic fibers woven into a fabric in a heart shape displaying color changes selectively.

These composite fibers could be continuously prepared for large-scale production, and Fig. 4a shows a spool of electrothermal chromatic fiber. The continuous fiber was stretched to 60% without any obvious degradation in the structure and electrothermal chromatic performance (Fig. 4b and c). Due to the intrinsic flexibility and stretchability, the electrothermal chromatic fibers could be woven into textiles or implanted into fabrics through the well-developed weaving technology. Fig. 4d and e show a fabric knitted from the electrothermal chromatic fibers which displayed a variety of symbols or signs by changing its colors. Additionally, the electrothermal chromatic fibers could also be implanted into clothes in a heart shape to change colors selectively (Fig. 4f–h). Importantly, the electrothermal chromatic fibers could be further deformed or stretched to adapt to body movement without fatigue in the chromatic performance (Fig. S13†).

## Conclusions

In conclusion, a flexible and stretchable chromatic fiber is developed by winding aligned CNT sheets and depositing PDA onto elastic fibers. The smart fibers exhibited reversible and distinct color changes, between dark purple and bright red, with the use of an electric current. The electrothermal chromatic transition was reversible for over 1000 cycles even under bending with angles from 0 to 180° and stretching up to 80%. These smart fibers could be fabricated on a large scale and woven or implanted into various fabrics for sensing and displaying. This work also provides a new platform for the real application of PDA-based materials.

## Supplementary Material

Supplementary information
