# Supplementary material for: Flexible and stretchable chromatic fibers with high sensing reversibility
Source: Chem Sci. 2016 Apr 14;7(8):5113–7. doi: 10.1039/c6sc00414h (PMC6020123; doi:10.1039/c6sc00414h)
Supplement: Supplementary file 1 [file SC-007-C6SC00414H-s001.pdf]

Electronic Supplementary Material (ESI) for Chemical Science.
This journal is © The Royal Society of Chemistry 2016

Supplementary Information

Flexible and Stretchable Chromatic Fibers with High Sensing Reversibility

Xin Lu, Zhidong Zhang, Xuemei Sun, Peining Chen, Jing Zhang, Hui Guo, Zhengzhong Shao* and Huisheng Peng*

State Key Laboratory of Molecular Engineering of Polymers, Department of Macromolecular Science, and Laboratory of Advanced Materials, Fudan University, Shanghai 200438, China; E-mail: sunxm@fudan.edu.cn, zzshao@fudan.edu.cn.

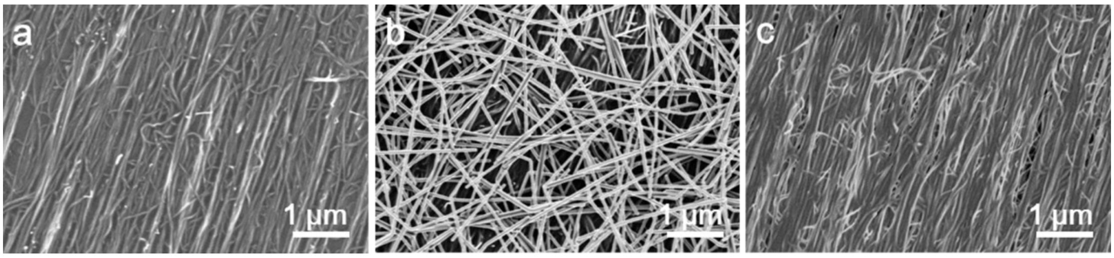

Fig. S1 SEM images of the surface of the conductive fiber during preparation. **a.** The inner CNT layer. **b.** The middle silver nanowire. **c.** The outer CNT layer.

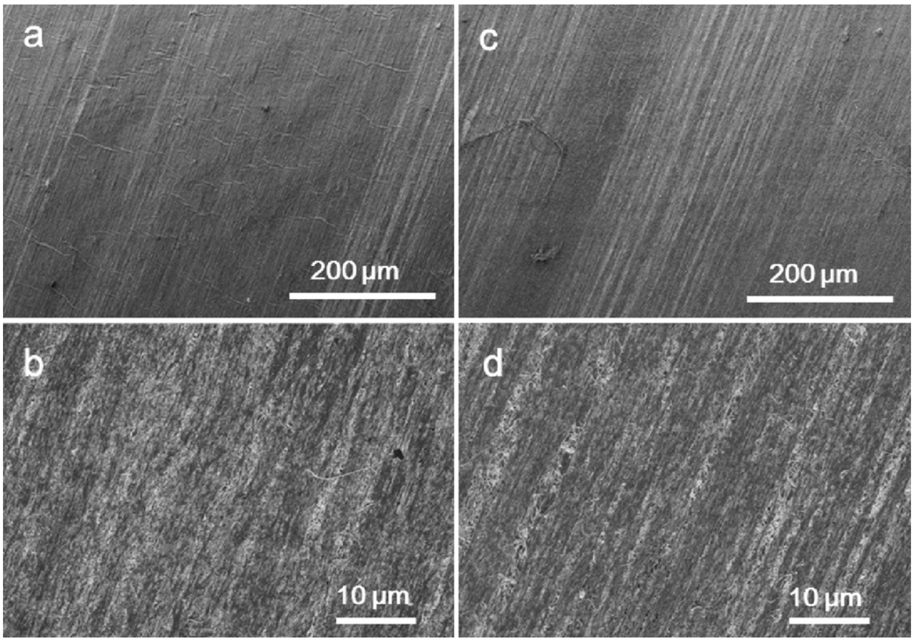

Fig. S2 SEM images of the elastic conductive fiber after stretching at different magnifications. **a** and **b.** Original state without stretching. **c** and **d.** After stretching (strain of 50%) for 1,000 cycles.

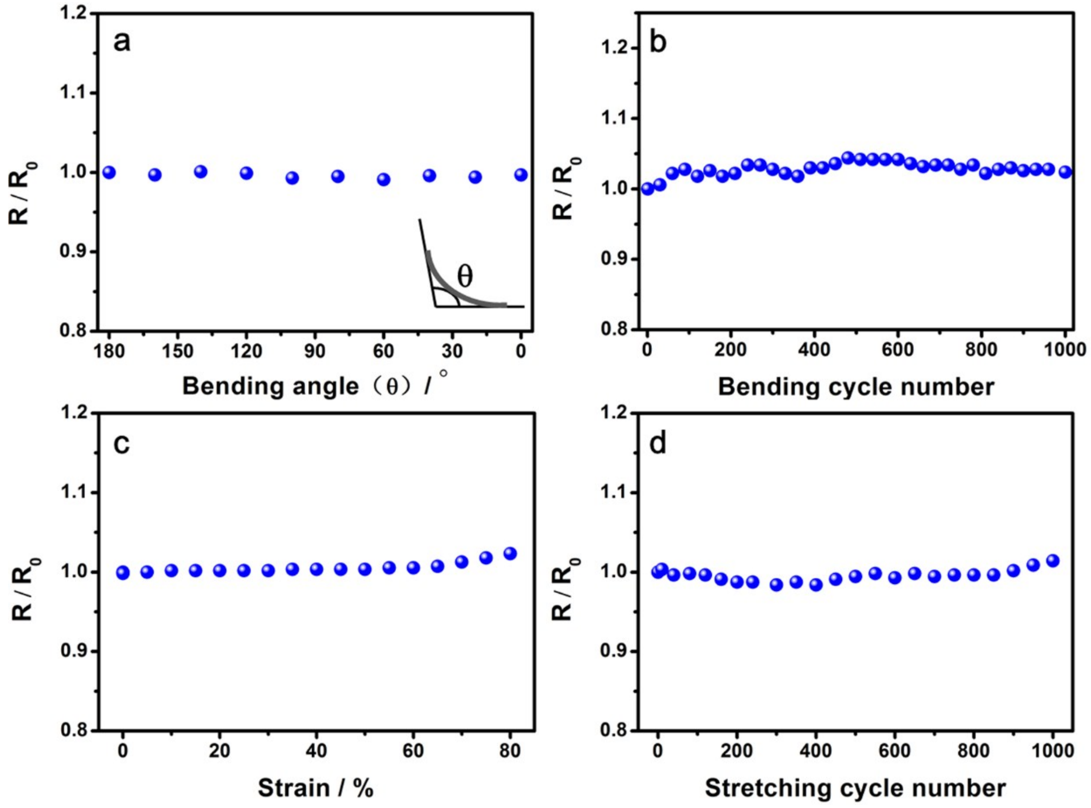

Fig. S3 Electrical property of the conductive fiber before and after bending or stretching. **a.** Dependence of resistance ratio on bending angle. **b.** Dependence of resistance on bending cycle number (with a bending angle of 90˚). **c.** Dependence of resistance ratio on strain. **d.** Dependence of resistance ratio on stretching cycle number (with a strain of 50%). Here R_0 and R correspond to the resistances before and after bending or stretching, respectively.

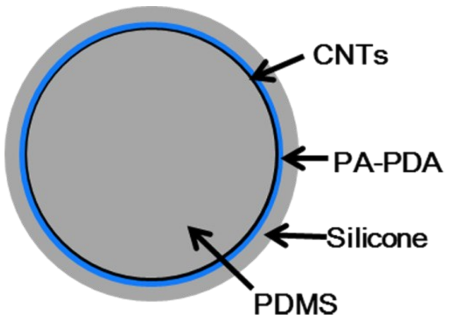

Fig. S4 Schematic illustration for the cross-sectional structure of the electrothermal chromatic fiber.

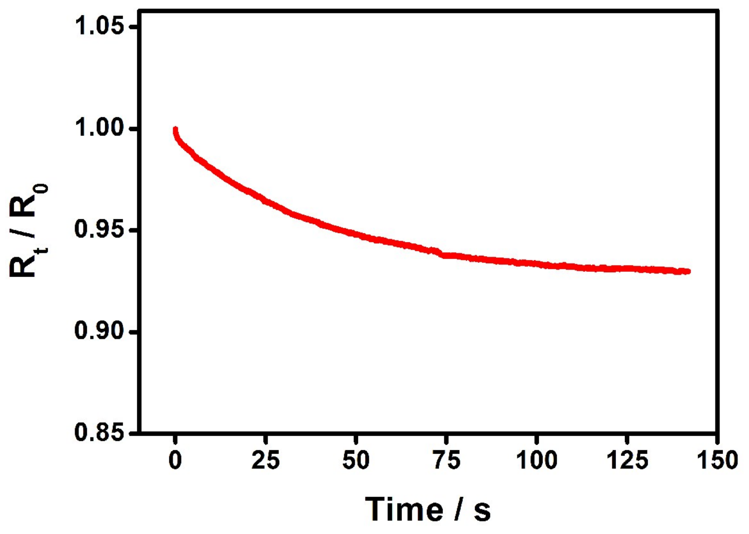

Fig. S5 The evolution of the resistance with time for the composite fiber at a current of 8.1 mA.

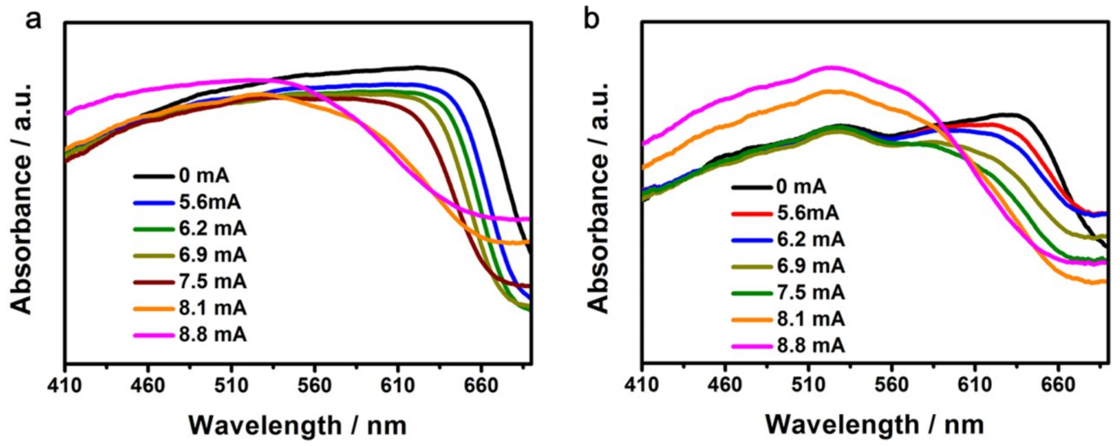

Fig. S6 Electrothermal chromatic performance of the fiber. **a.** UV-vis spectra of the fiber with increasing electric currents at the first cycle. **b.** UV-vis spectra of the fiber at increasing electric currents at the second reversible cycle.

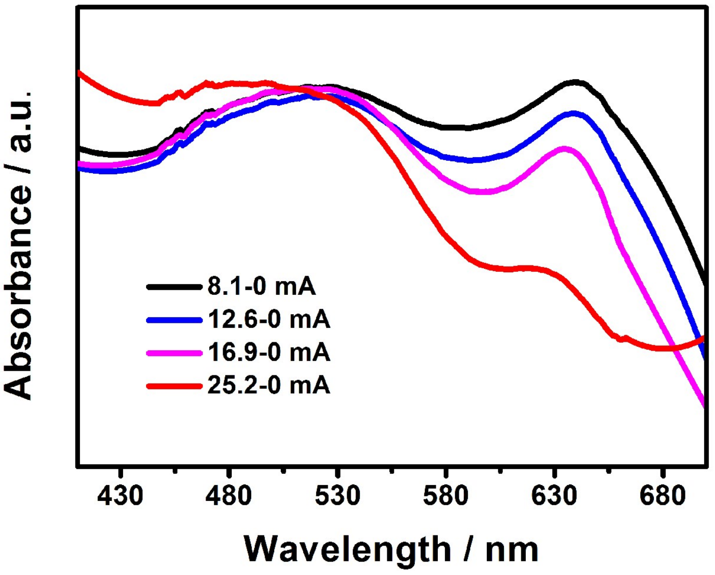

Fig. S7 UV-vis spectra of the electrothermal chromatic fiber after passing with currents of 8.1, 12.6, 16.9 and 25.2 mA.

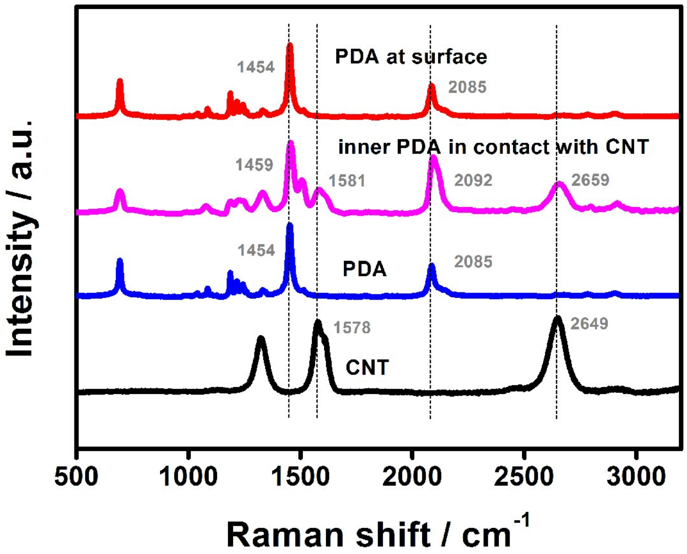

Fig. S8 Raman spectra of different locations in the composite PDA/CNT fiber in comparison to the bare CNT and bare PDA.

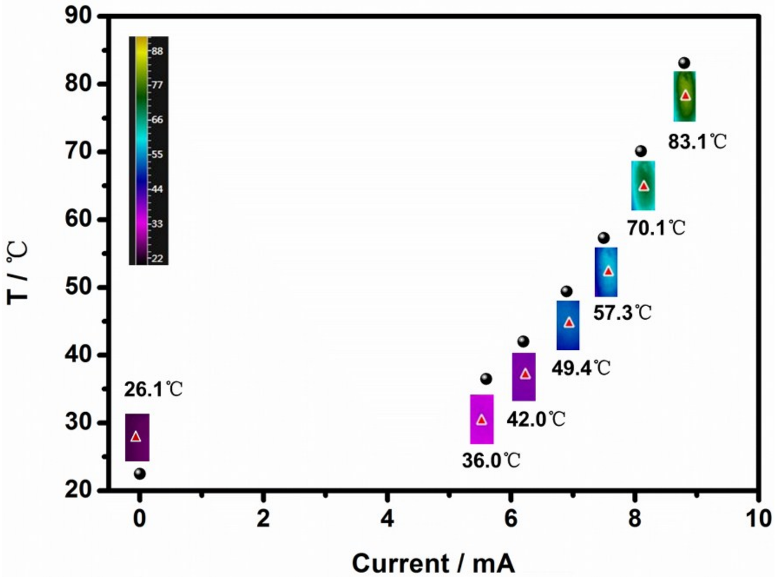

Fig. S9 Temperatures of the composite fiber with increasing electric currents.

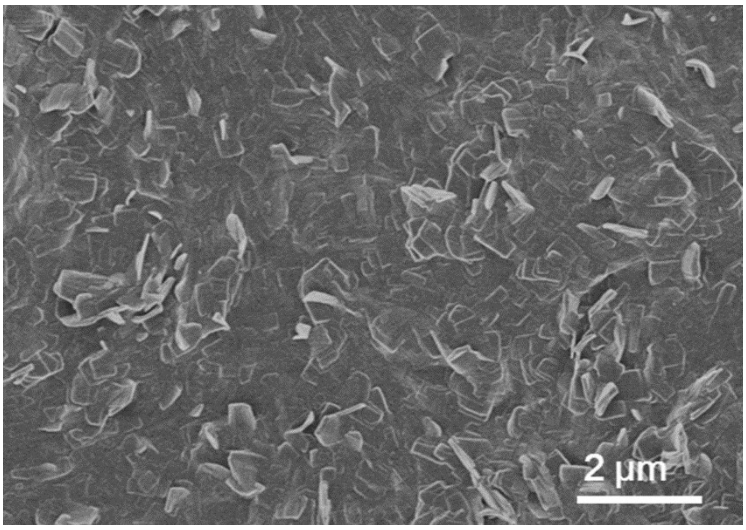

Fig. S10 Morphology transition of bare PDA after a heating and cooling process.

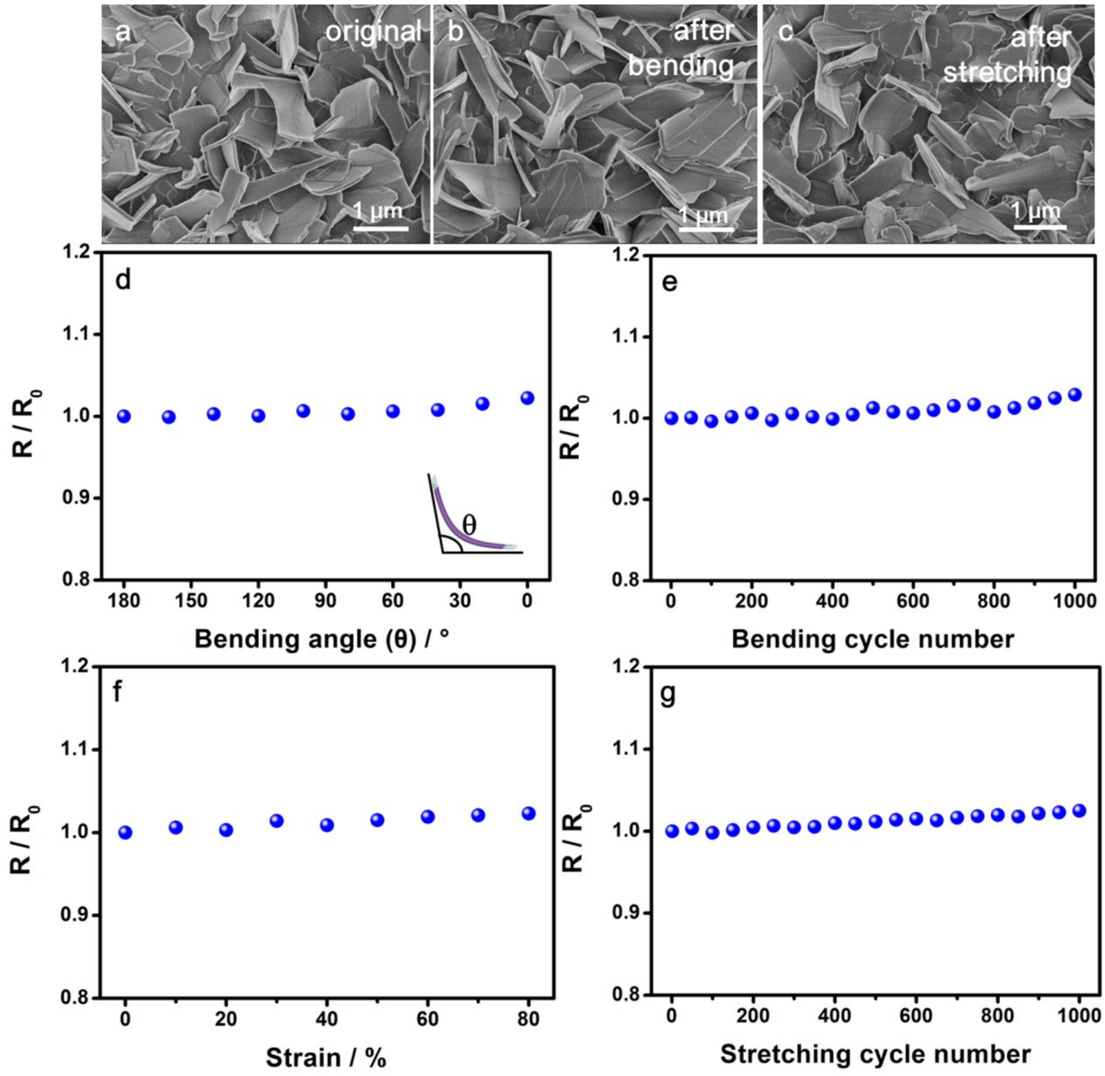

Fig. S11 The evolution in morphology and electrical property of the electrothermal chromatic fiber before and after bending or stretching. **a.** SEM image of an as-synthesized composite fiber. **b.** SEM image after bending. **c**. SEM image after stretching. **d.** Dependence of resistance ratio on bending angle. **e.** Dependence of resistance ratio on bending cycle number (with a bending angle of 90°). **f.** Dependence of resistance ratio on stretching. **g.** Dependence of resistance ratio on stretching cycle number (with a strain of 50%). Here R_0 and R correspond to the resistances before and after bending or stretching, respectively.

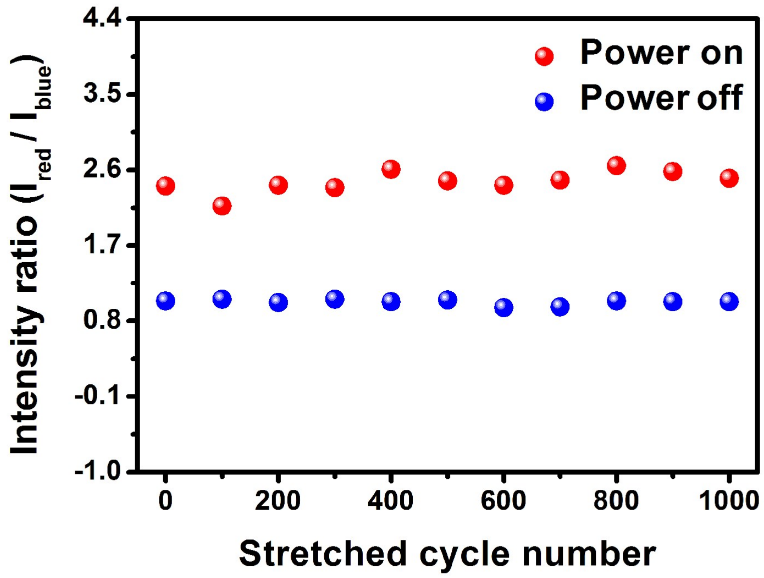

Fig. S12 Intensity ratio of red to blue peaks for a composite fiber being stretched by 50% for 1,000 cycles. The data were collected from UV-vis spectra.

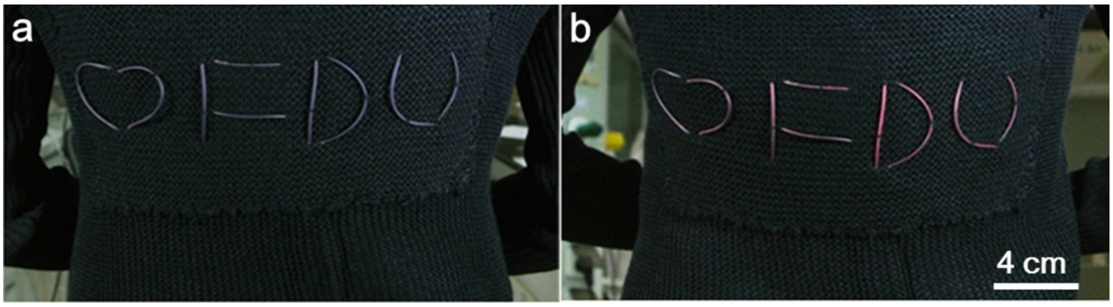

Fig. S13 Electrothermal chromatic fibers being woven into a sweater to display different colors.
